# An Explainable Deep Learning Model to Prediction Dental Caries Using Panoramic Radiograph Images

**DOI:** 10.3390/diagnostics13020226

**Published:** 2023-01-07

**Authors:** Faruk Oztekin, Oguzhan Katar, Ferhat Sadak, Muhammed Yildirim, Hakan Cakar, Murat Aydogan, Zeynep Ozpolat, Tuba Talo Yildirim, Ozal Yildirim, Oliver Faust, U. Rajendra Acharya

**Affiliations:** 1 Faculty of Dentistry, Department of Endodontics, Firat University, Elazig 23119, Turkey; 2 Department of Software Engineering, Firat University, Elazig 23119, Turkey; 3 Department of Mechanical Engineering, Bartin University, Bartin 74100, Turkey; 4 Department of Computer Engineering, Malatya Turgut Ozal University, Malatya 44700, Turkey; 5 Faculty of Technology, Department of Electrical-Electronics Engineering, Firat University, Elazig 23119, Turkey; 6 Faculty of Dentistry, Department of Periodontology, Firat University, Elazig 23119, Turkey; 7 Department of Computer Science, Anglia Ruskin University, Cambridge CB1 1PT, UK; 8 International Research Organization for Advanced Science and Technology (IROAST), Kumamoto University, Kumamoto 860-0811, Japan; 9 Department of Bioinformatics and Medical Engineering, Asia University, Taichung 41354, Taiwan; 10 School of Science and Technology, Singapore University of Social Sciences, Singapore 599494, Singapore; 11 School of Business (Information Systems), Faculty of Business, Education, Law & Arts, University of Southern Queensland, Toowoomba, QLD 4350, Australia

**Keywords:** caries, dental health, explainable deep models, deep learning, Grad-CAM

## Abstract

Dental caries is the most frequent dental health issue in the general population. Dental caries can result in extreme pain or infections, lowering people’s quality of life. Applying machine learning models to automatically identify dental caries can lead to earlier treatment. However, physicians frequently find the model results unsatisfactory due to a lack of explainability. Our study attempts to address this issue with an explainable deep learning model for detecting dental caries. We tested three prominent pre-trained models, EfficientNet-B0, DenseNet-121, and ResNet-50, to determine which is best for the caries detection task. These models take panoramic images as the input, producing a caries–non-caries classification result and a heat map, which visualizes areas of interest on the tooth. The model performance was evaluated using whole panoramic images of 562 subjects. All three models produced remarkably similar results. However, the ResNet-50 model exhibited a slightly better performance when compared to EfficientNet-B0 and DenseNet-121. This model obtained an accuracy of 92.00%, a sensitivity of 87.33%, and an F1-score of 91.61%. Visual inspection showed us that the heat maps were also located in the areas with caries. The proposed explainable deep learning model diagnosed dental caries with high accuracy and reliability. The heat maps help to explain the classification results by indicating a region of suspected caries on the teeth. Dentists could use these heat maps to validate the classification results and reduce misclassification.

## 1. Introduction

Oral diseases are estimated to affect approximately half of the world’s population today, of which 2.3 billion people worldwide suffer from permanent dental caries [1]. Dental caries, often known as tooth decay, is a disease characterized by tooth damage produced by bacteria in the mouth that create lactic acids, which directly harm the tooth’s enamel layer. This can eventually lead to a small gap between the teeth, which can cause pain, infection, and tooth loss if left untreated [2,3,4]. Not all caries lesions can be identified visually and tactilely. Therefore, imaging techniques are frequently used to improve the detection rate [5,6,7]. However, even with medical imaging, dentists may miss early caries lesions. Therefore, diagnosis and treatment success depends on the technology and individual performance of the reading expert [8]. Furthermore, examining X-ray images adds to doctors’ workload. An automatic detection approach with excellent consistency and accuracy is necessary to assist clinical stomatologists with objective caries diagnosis.

The visible light transillumination method [9], calibrated diaphragm computed tomography [10,11], international caries detection and assessment system [12], and quantitative light-induced fluorescence were all used in early works on dental caries detection. Another study established a system of improving panoramic dental radiology for diagnosing dental caries using image processing techniques [13]. Researchers also investigated computer-assisted caries detection with X-ray images [14,15,16,17]. With the Logicon Caries Detector (LCD), using upgraded CAD software, Tracy et al. [14] investigated and confirmed the efficiency of a density analysis auxiliary tool in helping dentists detect and classify caries based on user feedback. Using an early dentinal caries diagnostic tool, dentists could identify twice as many early dentinal caries. Oliveira et al. [15] designed a feed-forward artificial neural network to identify dental caries and classify whether there was caries in panoramic dental X-ray images. In their study, a classification accuracy of 98.7% was achieved. Osterloh and Viriri [16] developed an unsupervised learning approach through blob detection and cluster analysis for caries that performed well with an accuracy of 96% in diagnosis. Tikhe et al. [17] developed an algorithm for detecting enamel and interproximal caries utilizing digital periapical radiography images. Our analysis shows that no segmentation was conducted in any of these studies; only caries detection was performed. Caries segmentation is important because it provides further information regarding the degree of caries, such as the caries area, which may be required for further caries classification.

Deep learning has recently been a popular topic in dentistry, particularly in dental caries detection [18,19,20]. However, because there is little research concentrating on deep learning-based caries segmentation, it remains a topic that demands further attention in the field. Xu et al. proposed a 3D tooth segmentation method using deep convolutional neural networks (CNNs) [21]. Their mesh labelling method outperformed current geometry-based methods, with an accuracy of 99.06% measured by area. However, their method is limited when the boundary between two teeth is corrupted by the simplification procedure described in their approach. A large fraction of the error is due to the different appearances of wisdom teeth on each dental mesh. This is because wisdom teeth are either absent, less, or only partially visible. Since wisdom teeth data are so limited, the training stage is challenging. In 2022, researchers conducted caries segmentation using a U-shaped network and vision transformer, dilated convolution, and feature pyramid fusion methods on clinically collected tooth X-ray images [22]. The method was successful compared to UNet, Trans-Unet, and Swin-Unet, with an average dice similarity of 75% and an average pixel classification precision of 74% on the test set. However, due to clinical collection constraints, their analysis was limited by the small number of images in the dataset. Lee et al. [23] also conducted a similar study for early dental caries segmentation using Unet on bitewing radiographs. Their method also suffers from a lack of labelled data. Data augmentation techniques may be useful to improve the accuracy of deep learning-based approaches for classifying teeth by up to 5% [24]. All these studies focus on improving caries detection performance. However, the systems do not explain how these results are achieved. Hence, it is difficult for dentists to verify the outcomes, resulting in low trust in these systems. Recently, explainable artificial intelligence models have become interesting research fields [25].

This paper proposes an explainable deep learning-based method for computer-assisted automatic caries detection. The deep learning approach has identified various popular pre-trained networks based on CNN. In total, 13,870 caries and non-caries tooth images were obtained from 562 participants for network training and performance evaluation. We explain our results by overlaying a heatmap on the images. This heatmap allows human experts to verify the caries detection results, which might lead to higher levels of trust.

The main contributions of this study are as follows:

- End-to-end caries detection is achieved without the need for any feature extraction.
- The proposed deep learning model has an explainable structure with the Grad-CAM method.
- Our explainable structure showed that caries markings on the tooth regions during the decision phase help experts during the diagnosis phase.
- The performance of the model is evaluated by various performance metrics, and individual results are explained through heat maps.
- It is provided to mark the caries regions without needing any segmentation process.
- Our explainable model performs as efficiently as expert dentists in detecting and localizing caries.

## 2. Materials and Methods

**Figure 1:**
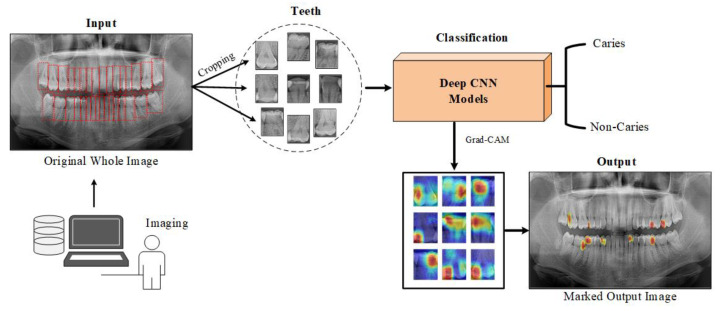
A block representation of the material and method used in the study.

This study proposes a deep learning model for detecting caries and non-carious teeth from panoramic dental images. Popular pre-trained CNN models are used to lower the expenses of model training from scratch. These models include pre-learned and optimized weights on large datasets, such as edges and shapes in images. The single tooth image given as input to the deep learning model is estimated as caries or not caries at the output. Heat maps are constructed using the Grad-CAM method to highlight which areas on the dental image are concentrated in the predictions of the CNN model. Figure 1 shows a block diagram of the proposed approach.

### 2.1. Dental Dataset

The dataset used in this study was obtained from 562 subjects with ethical permission (Id: 2022/03-18) from the Department of Firat University Faculty of Dentistry, Elazig, Turkey. Data were labelled caries and non-carious based on a specialist’s determination of tooth areas in panoramic tomography images. The specialist manually cropped the tooth regions in the panoramic tomography image. During the manual cropping process, a bounding box was defined for each tooth, and the expert adjusted the size and coordinates of the bounding box. Each bounding box’s area was then saved as a separate image.

**Figure 2:**
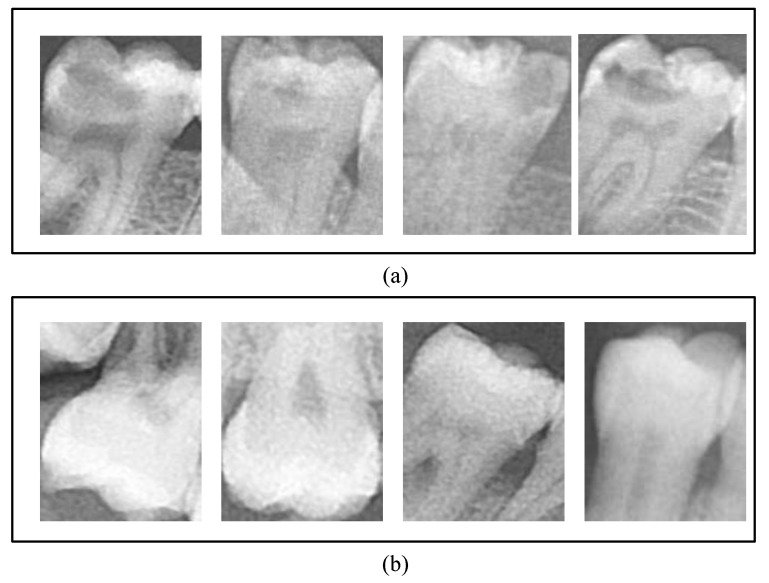
Demonstration of tooth samples used in our study: (**a**) Caries teeth and (**b**) non-caries teeth.

A total of 1160 caries and 1040 non-caries tooth images were obtained from all panoramic images. In Figure 2, tooth samples with caries and non-carious labels are provided.

Data augmentation techniques have been used to prevent the negative effects that may occur due to the small number of images. The data augmentation procedure utilized rotation, shift, shear, zoom, flip, and fill. Augmentation was applied to the training and validation sets but not to the test set. Using the data augmentation method, the number of caries and non-caries samples in the training and validation phase increased to 6635. Therefore, a total of 13,870 tooth samples were employed for training and validation within the scope of this study. We used 300 caries and 300 non-caries images during the test phase. These images were selected from different subjects used during the training and validation phase. Table 1 provides detailed information on the numerical distribution of dataset samples following data division.

### 2.2. Proposed Classification Method

**Figure 3:**
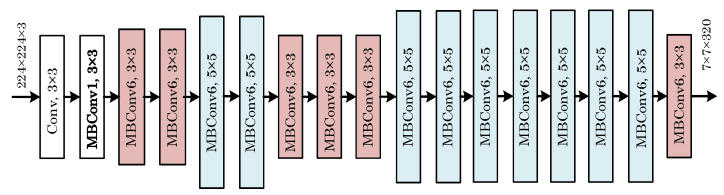
A block representation of the EfficientNet-B0 deep learning model.

This study employed CNN-based EfficientNet-B0 [26], DenseNet [27], and ResNet [28] models for the classification process. The stated models’ performance has already been established successfully after they were trained on large image datasets. These models are pre-trained models since they were previously trained, and their weight has been optimized. These weights can be used in other models by using the transfer learning method. The last layers of the deep learning models mentioned in this study were retrained for tooth images and used in the study. Given the challenges of developing problem-specific solutions, the models were trained on large datasets, which can benefit a wide range of image classification studies. CNNs are typically built at a fixed resource cost, and then the number of layers is expanded when more resources become available to improve the performance. Increasing the number of layers results in a higher input resolution for training and evaluation, which can enhance the model’s accuracy. However, even if the mentioned methods increase the model accuracy, their performance may remain insufficient. To address that issue, Tan et al. [26] proposed a new model scaling technique that uses the combined coefficient to scale CNNs in a structured manner. Their method used fixed-size scaling instead of increasing the number of layers. The EfficientNet model family is smaller and faster than other models because it is based on a novel scaling method. EfficientNet-B0 is the base model, and EfficientNet-B1 to EfficientNet-B7 have scaled variants of the base model. Figure 3 shows a block diagram of EfficientNet-B0.

**Figure 4:**
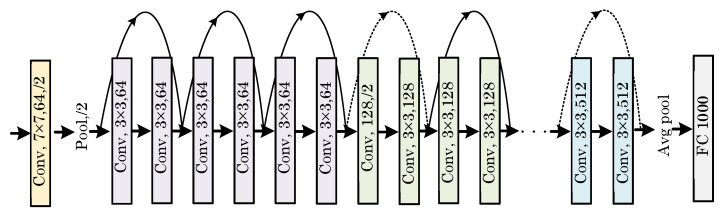
A block diagram representation of the ResNet model depicting its working structure.

He et al. [28] introduced a deep learning model called ResNet, which won the ILSVRC ImageNet competition in 2015. The main difference between ResNet and previously proposed architectures is that it has a more complex structure. Batch normalization was utilized for the first time in a Deepnet model. Some versions of this architecture consist of 26 million parameters, including Resnet18, Resnet50, and Resnet101. Figure 4 shows a block depiction of the ResNet model structure.

**Figure 5:**
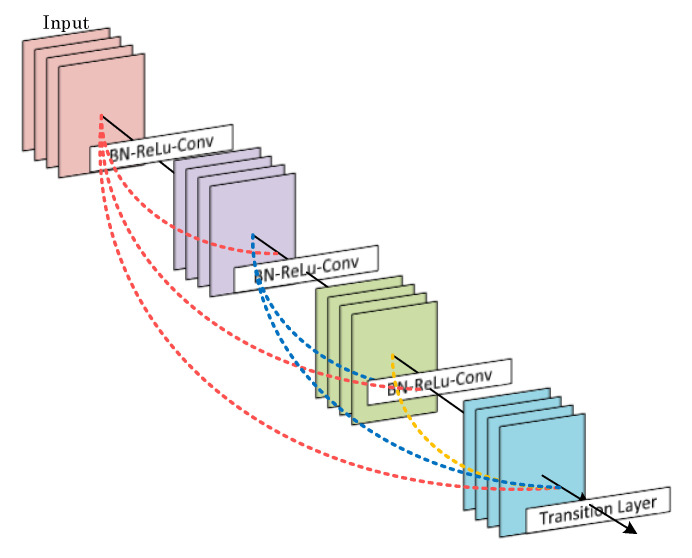
A block diagram representation of the DenseNet deep learning model.

Huang et al. [27] developed a deep learning model where each layer is connected to all other layers. Due to the dense connections between the layers, it is termed DenseNet. Such densely connected architectures use feature maps of previous layers as the input for each layer. In addition, each layer’s feature maps are used as inputs for each subsequent layer. In the DenseNet design, each layer keeps the original data and the activation from the previous layers. The model is denser and more efficient because it has shorter connections between the input and output layers. A visual representation of the DenseNet model’s working structure is shown in Figure 5.

## 3. Experiments

### 3.1. Experimental Setup

The deep learning model was trained using the EfficientNet-B0, DenseNet-121, and Resnet-50 models. The Keras library, designed for the Python programming language, allows models with a different number of layers to be included in the training process. We used ImageNet weights instead of random starting weights in the training models. The relevant models’ final layers have been customized to differentiate between caries and non-caries images. In the redesigned layers, Softmax was employed as an activation function. We chose Adam optimization, cross-entropy loss, 16 batch size, and a learning rate of 0.001, and the early stop function was active for 50 epochs to obtain the highest performance. All these operations took place in a Google Colab environment. The early stop feature was activated, and the weights with the highest value were recorded when the validation accuracy rate did not exceed the highest value for five consecutive rounds. We resized the tooth images before they were used for training and testing due to their poor resolution. To determine the most suitable model EfficientNet-B0, DenseNet-121, and ResNet-50 models were trained with the same hyperparameters. After completing the training phases, several performance measures were evaluated, and the best classifier model was identified.

### 3.2. Performance Evaluation Metrics

In classification studies, confusion matrix-based performance measures are utilized to assess the performance of the deep learning model. The confusion matrix shows the relationship between the deep learning model’s predicted class label for the input image in the output layer and the input image’s ground-truth label. Binary classification models can predict two classes at the output. As a result, there can be up to four possible scenarios in class estimation. The following are the details of these cases [29,30]:

- The first case, True Positive (TP), occurs when the classifier’s deep learning network predicts that an image with a Caries label has Caries.
- The second case, False Positive (FP), occurs when the classifier’s deep learning network predicts that an image with no Caries label has Caries.
- The third case, False Negative (FN), is when the classifier deep learning model predicts an image with a Caries label as no Caries.
- The fourth case, called True Negative (TN), is when an image known to have no Caries label is predicted as no Caries by the classifier deep learning model.

The confusion matrix is assembled by placing the TP, FP, TN, and FN values in a 2 × 2 matrix. A high number of TP and TN cases indicates a good classification performance. TP, FP, TN, and FN values have been used to standardize several performance metrics for measuring classification performance. These measures are listed below, along with their mathematical definitions.

Accuracy = (TP + TN)/(TP + FP + FN + TN)(1)

Sensitivity = TP/(TP + FN)(2)

Precision = TP/(TP + FP)(3)

Specificity = TN/(TN + FP)(4)

F1 Score = (2 × (Precision × Sensitivity))/((Precision + Sensitivity))(5)

Matthews Correlation Coefficient (MCC) = ((TP × TN) (FP × FN))/√((TP + FP)(TP + FN)(TN + FP)(TN + FN))(6)

### 3.3. Results

**Figure 6:**
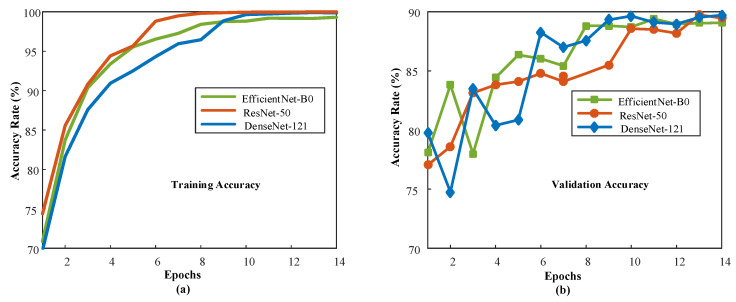
Model performance curves during training: (**a**) Training accuracy values for all models, (**b**) validation accuracy values for all models.

**Figure 7:**
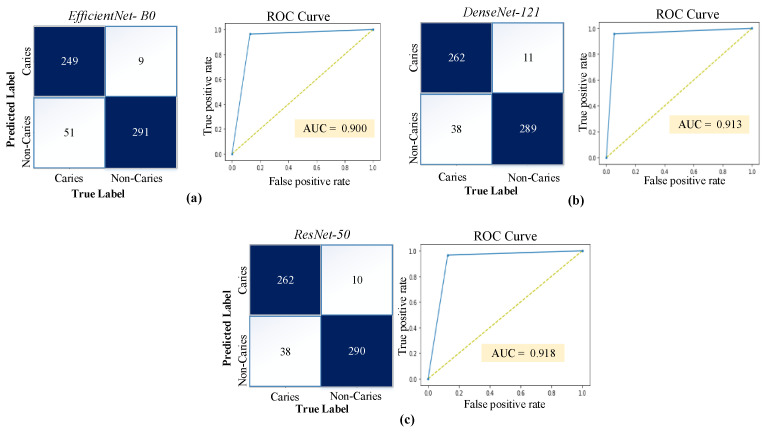
Confusion matrices and ROC curves obtained by the models on the test images: (**a**) EfficientNet-B0, (**b**) DenseNet-121, and (**c**) ResNet-50.

Each model was trained separately, and the number of training epochs was adjusted according to the early stop condition. Figure 6 documents the training process by plotting the model performance over the training epochs. After the first training epoch, the models achieve 70% classification accuracy and above. The good performance during the initial training epoch indicates that the pre-trained network layers could extract relevant information from the dental images. Figure 7 shows the confusion matrices and ROC curves for each model.

Table 2 documents the best performance of each model during the training phase. The accuracy values obtained for the test data are 90.00%, 91.83%, and 92.00% for the EfficientNet-B0, DenseNet-121, and ResNet-50 models, respectively. Despite the model’s performances being quite close to each other, the ResNet-50 model outperformed the others by a slight margin. The sensitivity, specificity, precision, F1-score, and MCC values of all models are also detailed in Table 2.

**Figure 8:**
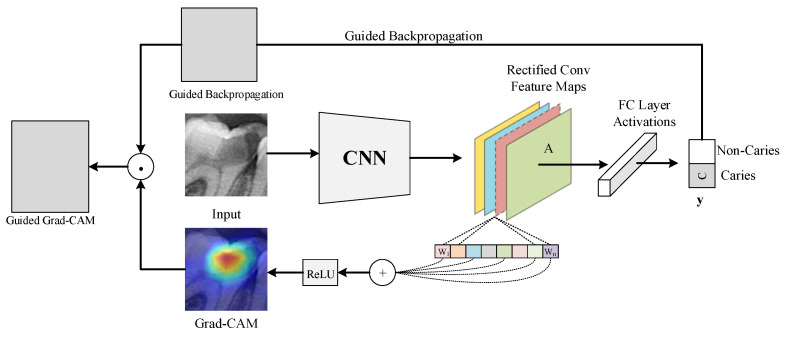
A block diagram representing the Grad-CAM algorithm’s working structure.

Numerical performance measures provide helpful information about the ability of a model to differentiate between caries and non-caries tooth images. However, these values do not explain how the results were obtained. This places serious restrictions on root cause analysis for failure case investigations. During such an investigation, we must treat the deep model results as coming from a black box because we cannot trace them in the deep network. In our case, this black box is described by the performance parameters. These parameters are not helpful during a single root cause analysis because the accuracy values indicate a non-zero chance of failure. Even the ResNet-50 is inaccurate in 8% of all cases. We have addressed that issue by documenting the image regions where a deep learning model focuses its efforts. The technique used was Grad-CAM [31], and the results were shown as heat maps. The heat maps help human experts to determine whether the areas in the images of a class are accurately predicted by the deep learning model while focusing on the areas that play an active part in determining the class. In Figure 8, the working framework of the Grad-CAM algorithm is depicted.

**Figure 9:**
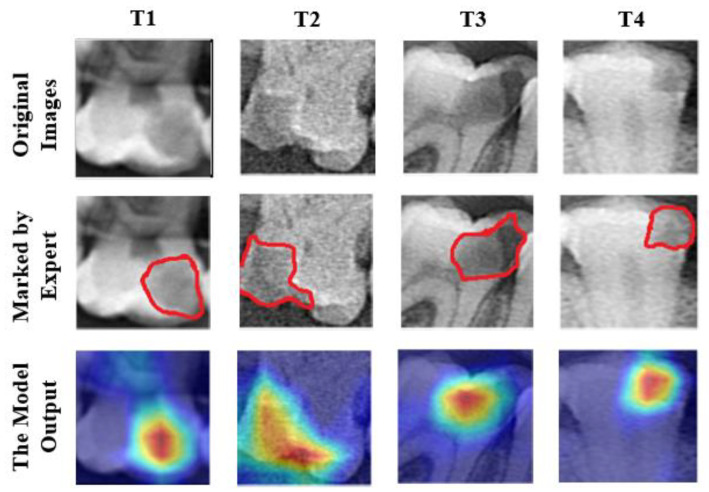
Heat maps of various test images were obtained using the Grad-CAM method. Red lines indicate the expert-marked caries regions.

The Grad-CAM algorithm performs its operations by referencing the feature maps produced by the last convolution layer of a CNN. As a result, the last convolution layer of the classifier deep learning model should be referenced with the Grad-CAM algorithm with its terminology. After using the last convolution layer as a reference, a heat map using gradients is created to emphasize the critical locations of the class label on the image. In the generated heat maps, the yellow and red areas indicate the pixel areas that the classifier’s deep learning network pays attention to while making predictions. In this study, Grad-CAM outputs of several test images are given in Figure 9. The test images were first sent to specialist dentists for marking the caries areas. The areas marked by dentists and the heat map regions generated by the model were compared.

**Figure 10:**
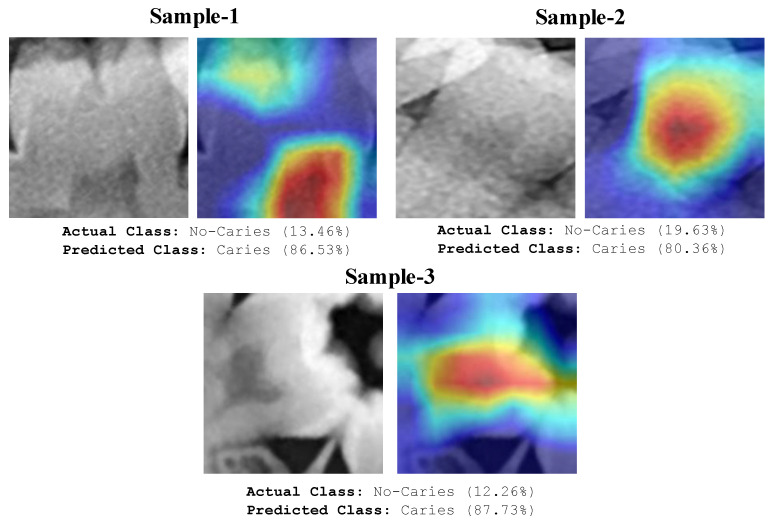
Original images and heatmaps of some test images misclassified by the proposed model.

The areas focused by the deep model and the areas marked by the expert overlap significantly, as seen in Figure 9. These images demonstrate that the deep learning model focuses on the correct regions and accurately predicts caries. In addition to accurate predictions, heat maps can provide crucial information about images that the model predicted wrongly. Some examples of tooth images misclassified by the model are shown in Figure 10.

**Figure 11:**
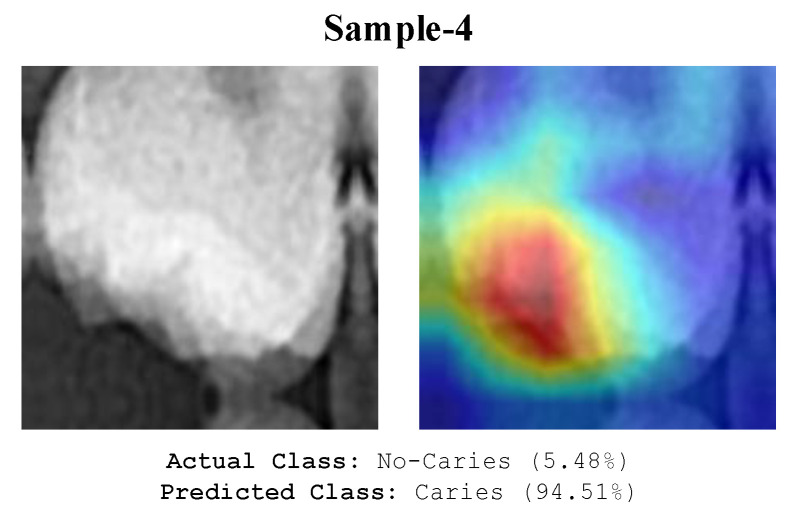
An illustration of the image that the model misclassified due to artifacts.

The model appears to focus on the pulp chambers shown in Figure 10. The model wrongly classified caries and the pulp chamber since the X-ray image focused near radiolucency. Even experts make similar errors. Depending on the anatomical nature of the tooth, caries-like artefacts may appear on the X-ray images in various locations. These artefacts can cause images to be misinterpreted by the model. The analysis of the image in Figure 11 misclassified by the model shows the formation of radiolucency in the focused area of the model due to the misalignment of the buccal and palatal cups of the tooth. The deep model also evaluated these areas as caries.

**Figure 12:**
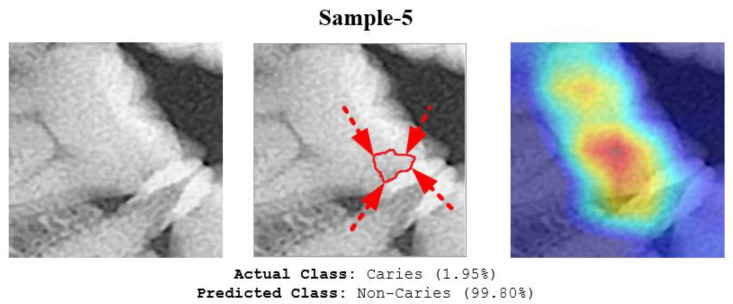
The proposed model misclassified the image of Sample 5. The red line and arrows indicate the early-stage caries area, as marked by the expert.

The deep model also misclassified the image sample as shown in Figure 12. One reason for this error might be that the area of initial-stage caries was not clearly radiolucent. Furthermore, it was too small and did not form cavitation. As a result, even a human expert might ignore this type of early-stage caries.

## 4. Discussion

Automated caries detection with medical images is an active research area. Table 3 provides details for a hand-curated selection of research studies on that topic. Most of the selected works used CNN-based pre-trained models for medical decision support. In their study, Singh and Sehgal used the CNN-LSTM architecture on the dataset containing 1500 images and achieved an accuracy value of 96%. They performed better than successful pre-trained models, such as GoogLeNet and Alexnet [32]. Salehi et al. [33] used a CNN model to predict enamel, dentin, and non-caries images on 81 samples. Huang and Lee [34] used ResNet-152 in their work and achieved an accuracy rate of 95.21% with 748 images. Lakshami and Chitra [35] detected cavities in teeth images with the AlexNet-based model. Their model yielded a 96.08% accuracy rate. Leo and Reddy [36] detected caries with an accuracy of 96.00% with the DNN model.

This study used a ResNet-50 pre-trained model for automated caries detection. The proposed approach yielded 92.00% accuracy. Leo and Reddy [36] achieved 96% accuracy with a Deep Neural Network (DNN) for a similar caries non-caries problem. However, their result was based on only 480 images whereas ours was based on 13,870 images. Hence, our network could extract knowledge from significantly more image data. This might lead to better generalization, which has advantages during the practical deployment of the model. All other works listed in Table 3 focused on slightly different problems making a direct comparison via performance measures infeasible. Huang and Lee [34] used ResNet-152 in their work and achieved an accuracy rate of 95.21% with 748 images. Lakshami and Chitra [35] detected cavities in teeth images with an AlexNet-based model. Compared to the studies listed in Table 3, we obtained higher accuracy and used a greater number of images.

**Figure 13:**
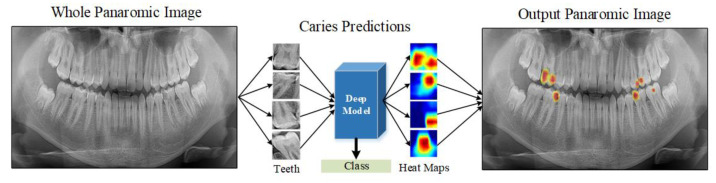
A clinical use case of the proposed caries detection model to automatically detect caries areas on the entire panoramic tooth image.

The advantages of our explainable model can be summarized as follows:

- Due to its explainable structure, it is simple to determine which areas the model focuses on during the decision phase. As a result, it can assist dentists in their decision-making.
- Our developed automated system can assist junior or trainee dentists as an adjunct tool to make an accurate diagnosis.
- Caries areas can be accurately determined using heat maps without any segmentation techniques.
- Clinical application of the software that can detect caries areas on the input panoramic radiography image is achievable using the proposed approach, as illustrated in Figure 13.

The limitations of the proposed approach can be summarized as follows. First, labelling issues may arise during the collection of caries images due to individual errors made by experts. In this study, whole panoramic radiography images were manually acquired. As a result, data collection is time-consuming. Moreover, we used images from only 562 subjects. In the future, we intend to increase the number of subjects and increase the number of classes. Furthermore, we will explore the possibility of using deep-learning-based approaches to detect teeth on whole panoramic radiography images automatically. The proposed model can be integrated into dental software and used in clinical settings.

## 5. Conclusions

In this study, a deep-learning-based explainable approach for the automatic detection of dental caries using panoramic radiography images from 562 subjects was proposed. Three popular pre-trained models were trained and tested. The ResNet-50 model yielded the highest accuracy of 92.00%, a sensitivity of 87.33%, and an F1-score of 91.61%. With this performance, it was the best among the tested models. The main limitation of this work is that we used images from only 562 subjects. The performance of the model can be improved by using images from more subjects. The Grad-CAM method was used to generate heat maps to visualize the regions upon which the model focused during the decision phase. Our results indicate that the model is highly reliable and focused on the correct areas during the decision-making stage. The proposed explainable deep learning model has the potential to be used in the early detection of dental caries, potentially leading to earlier treatment and better patient outcomes. The model may also be able to detect other dental problems, such as dental caries or periodontal disease, and could be used in conjunction with other diagnostic tools to provide a more comprehensive assessment of dental health.

## Figures and Tables

**Table 1:** The numerical distributions of the data used in the models’ training, validation, and testing stages.

Phase	Number of Original Data		Number of Augmented Data	
	Caries	Non-Caries	Caries	Non-Caries
Train	860	740	6635	6635
Test	300	300	300	300
Total	2200		13,870	

**Table 2:** Test data performance values obtained using various models employed in this study.

Deep Model	Accuracy (%)	Sensitivity (%)	Specificity (%)	Precision (%)	F1-Score (%)	MCC (%)
EfficientNet-B0	90.00	83.00	97.00	96.51	89.25	80.80
DenseNet-121	91.83	87.33	96.33	95.97	91.45	84.01
ResNet-50	92.00	87.33	96.67	96.32	91.61	84.37

**Table 3:** Comparison of our work with some state-of-the-art study techniques (deep learning) developed for automated caries detection.

Study	Number of Class	Number of Images	Classifier	Accuracy
Singh and Sehgal [32]	6 (Class I-VI)	1500	CNN-LSTM	96.00%
Salehi et al. [33]	3 (Non-caries, Enamel, Dentin)	81	CNN	90.75%
Wang et al. [37]	4 (Sound, White-spot lesions, Smashed, Plaque)	7200	T-Net CNN	95.45%
Huang and Lee [34]	3 (Non-caries, Enamel and Dentin)	748	ResNet-152	95.21%
Lakshmi and Chitra [35]	2 (Cavity, No cavity)	1900	AlexNet	96.08%
Leo and Reddy [36]	2 (Non-caries, Caries)	480	DNN	96.00%
Zhu et al. [38]	3 (Shallow caries, Moderate caries, Deep caries)	3127	CariesNet	93.61%
The proposed study	2 (Caries, Non-caries)	13,870	ResNet-50	92.00%

## Data Availability

Not applicable.
